# The novel functions of chemokines in lung cancer progression

**DOI:** 10.3389/fimmu.2025.1607225

**Published:** 2025-06-18

**Authors:** Xiaorui Gong, Xueying Wang, Jing Jin, Zhiqiang Gong

**Affiliations:** ^1^ Medical Oncology Department of Thoracic Cancer (II), Cancer Hospital of China Medical University Liaoning Cancer Hospital and Institute, Cancer Hospital of Dalian University of Technology, Shenyang, China; ^2^ Department of Child Healthcare, Shenyang Children’s Hospital, Shenyang, China; ^3^ Abdominal Radiotherapy Ward II, Cancer Hospital of China Medical University Liaoning Cancer Hospital and Institute, Cancer Hospital of Dalian University of Technology, Shenyang, China

**Keywords:** chemokines, lung cancer, immunotherapy, CCR9, CXCL12, PI3K pathway

## Abstract

Chemokines are key molecules that regulate immune cell migration and play critical roles in the tumor microenvironment. In lung cancer, chemokine dysregulation is closely linked to tumor progression. They promote immune cell infiltration and interact with tumor cells, enhancing tumor invasiveness and metastatic potential. This review highlights chemokine-mediated mechanisms, focusing on CCR9/CCL25 and CXCL12/CXCR4 axes, which promote tumor growth, metastasis, and immune evasion via PI3K/AKT and MAPK signaling. Elevated expression of these pathways correlates with poor outcomes and aggressive phenotypes. In SCLC, CXCR4 inhibitors show therapeutic promise when combined with chemotherapy or immunotherapy. This review summarizes the prognostic and therapeutic relevance of chemokines in lung cancer progression.

## 1 Introduction

Lung cancer remains the leading cause of cancer-related mortality worldwide, characterized by its high incidence, insidious onset, and poor prognosis (1). Lung cancer is notable for its rapid proliferation, early metastasis, and resistance to conventional therapies (2). Recent research has revealed that chemokines (3), small secreted proteins that regulate immune cell migration, exert dual roles in the tumor microenvironment (TME), acting as both promoters of tumor progression and modulators of antitumor immune responses (4). On the one hand, tumor-derived chemokines can recruit immunosuppressive cells such as regulatory T cells (Tregs), myeloid-derived suppressor cells (MDSCs), and tumor-associated macrophages (TAMs), thereby facilitating immune evasion, angiogenesis, and metastasis (5, 6). On the other hand, chemokines also orchestrate the trafficking of cytotoxic lymphocytes, dendritic cells, and other effector cells that mediate antitumor immunity (7). Among these, CCL25 and its receptor CCR9 (8), as well as CXCL12 and its receptor CXCR4 (9), have drawn significant attention due to their involvement in key oncogenic signaling pathways. These ligand-receptor axes not only mediate tumor cell survival and metastasis but also shape immune responses within the TME, making them promising targets for prognostic assessment and therapeutic intervention in lung cancer (10–13).

This review systematically summarizes the biological functions and molecular mechanisms of the CCR9/CCL25 and CXCL12/CXCR4 axes in lung cancer. Specifically, we describe in detail how chemokines influence proliferation, apoptosis, invasion, metastasis, and immune modulation via PI3K/AKT, MAPK, and other signaling pathways. Furthermore, we summarize current research progress on the clinical implications of chemokine expression and the emerging potential of chemokine-targeted therapies, particularly CXCR4 inhibitors, in improving patient outcomes.

## 2 Chemokines in regulation of NSCLC

CCR9, serves as the specific receptor for CCL25 (14), preferentially marks naive T cells and subsets of mature dendritic cells. Initially recognized for its role in recruiting immune cells, and act as a homing marker for lymphocytes involved in inflammatory responses, immune reactions, and leukocyte regulation, also influencing the chemotactic activity of CD4^+^ T lymphocytes (15). Studies have indicated that the organ-specific metastasis of tumors is associated with CCR9/CCL25, which is overexpressed in various malignancies, impacting patient prognosis (14, 16).

The PI3K/AKT signaling pathway is integral to the regulation of cellular proliferation throughout the body, primarily through the activation of signals from specific ligand-receptor interactions that inhibit apoptosis (17, 18). Normally, this pathway starts with PI3K, with AKT as a crucial hub, phosphorylating and thereby activating or inhibiting downstream targets such as EGFR, BCL-2, VEGFR, and MMP-9 to ensure normal cell proliferation and growth differentiation (19–21). In malignant tissues, aberrations such as amplification of PI3K, hyperactivation of AKT, or loss of regulatory factors can disrupt the balance of the PI3K/AKT pathway, leading to cancer cell invasion and metastasis (22, 23). In NSCLC, the PI3K subunit p85α is overexpressed and silencing the chemokine receptor CCR9 can reduce CCL25-induced PI3Kp85 and p-AKT production (24, 25). Activation of AKT, observable in transformed bronchial epithelial cells, induces malignant lung tumors, with higher AKT expression correlating with poorer prognoses in NSCLC patients. Studies by Li (24) also demonstrate that high expression of activated AKT in cancer tissues of NSCLC patients suggests a significant role of the PI3K pathway in the pathogenesis and progression of lung cancer (26).

### 2.1 Regulation of NSCLC proliferation and apoptosis by CCR9 and CCL25

Li (24) demonstrated that the CCR9/CCL25 axis facilitates the transition of cells from G1 to S phase, maintaining cell cycle continuity. Disruption of this axis results in decreased expression of cyclins in NSCLC tumor cells (27). It has been shown through the use of PI3K inhibitors that blocking the CCR9/CCL25 axis ensures the continuity of the cell cycle, upregulates cyclin expression, and activates PI3K and AKT. BCL-2, a downstream protein of AKT, promotes apoptosis by binding with Bad; however, activated AKT can prevent the association of Bad with BCL-2, thereby inhibiting apoptosis. Additionally, the anti-apoptotic factor BCL-2 can prevent the activation of Caspase-3 by releasing cytochrome C, thus blocking apoptosis. Another apoptotic factor, Survivin, under the downstream of BCL-2, blocks the Caspase-3 protease directly, exerting an anti-apoptotic effect. Expression levels of BCL-2 in lung cancer tissue are consistent with those of survivin (28). Sharma and Li (28, 29)found that inhibiting the CCR9/CCL25 axis could downregulate proteins such as PI3K, AKT, ERK1/2, and GSK-3β, while upregulating Caspase-3, thereby driving apoptosis. Zhong (30) research indicates that the expression of CCR9 in NSCLC correlates with BCL-2 and Survivin, suggesting that CCR9’s role in NSCLC may be through the inhibition of apoptosis.

### 2.2 CCR9 and CCL25 in NSCLC invasion and metastasis

From pathological and physiological perspectives, both angiogenesis (the formation of new blood vessels) and lymphangiogenesis (the formation of new lymphatic vessels) are essential for tumor maturation and lymph node metastasis. Vascular endothelial growth factor (VEGF), primarily located in the cytoplasm of cancer cells, not only induces endothelial cell mitosis to promote angiogenesis and lymphangiogenesis but also enhances plasminogen and collagenase secretion, increasing vascular permeability to support tumor nutrition. Niu (27) found that VEGF expression in peri-tumoral tissues exceeds that in tumor tissues, with its positivity rate closely linked to pathological types and lymph node metastasis. VEGF expression is higher in tissues with large lymph node metastases (≥1 cm) than in those with small metastases (≤1 cm), making it a valuable marker for assessing tumor malignancy, differentiation, and metastatic potential. VEGF also binds to Vascular Endothelial Growth Factor Receptor 3, promoting lymphangiogenesis via the MAPK and PI3K pathways (27, 31).

Matrix metalloproteinases (MMPs), particularly MMP-2 and MMP-9, play a critical role in tumor metastasis by degrading the extracellular matrix. Overexpression of MMP-9 in lung adenocarcinoma is associated with lymph node metastasis and is an independent risk factor for patient survival (32, 33). Li et al. demonstrated that the PI3K/AKT pathway drives MMP-2 expression in malignant tumors (34), while Lee et al. showed that activated AKT increases MMP-9 and MMP-2 expression, enhancing cancer cell invasion (35). Gupta (36) reported that MMP-2 promotes CCR9/CCL25 interactions, facilitating lung cancer cell invasion and selective metastasis. MMP-2 exhibits greater reactivity to CCL25 in lung adenocarcinoma compared to lung SCC, likely due to differential MMP activity and CCR9 phosphorylation (36). In normal tissues, MMPs and tissue inhibitors of matrix metalloproteinases (TIMPs) maintain extracellular matrix balance. However, in malignant tumors, activated MMP-2 and MMP-9 degrade the matrix, promoting cancer cell infiltration. While TIMPs were initially considered anti-cancer proteins, Bourboulia found that TIMP-2 overexpression stimulates lung adenocarcinoma cell proliferation (37–39). Gupta further revealed that lung adenocarcinoma cells produce TIMP-1 and TIMP-2, whereas lung SCC cells only express TIMP-2, suggesting that MMP and TIMP differential expression, mediated by the CCR9/CCL25 axis, influences lung cancer metastasis and invasion (36).

### 2.3 Regulation of NSCLC via the JAK/STAT and NF-κB pathways by chemokines

In addition to the PI3K/AKT and MAPK signaling pathways, chemokines also exert their oncogenic effects in lung cancer through modulation of the JAK/STAT and NF-κB signaling cascades. The JAK/STAT pathway, a critical mediator of cytokine signaling, has been implicated in tumor immune evasion, proliferation, and survival. Studies have shown that CXCL12/CXCR4 activation leads to downstream STAT3 phosphorylation, promoting transcription of genes related to tumor growth and angiogenesis, such as VEGF, MCL-1, and BCL-XL. Overactivation of STAT3 contributes to resistance against apoptosis and enhances immune suppression within the tumor microenvironment. In NSCLC, aberrant CXCL12/CXCR4-JAK/STAT3 signaling has been associated with poor clinical outcomes and therapeutic resistance. Similarly, the NF-κB signaling pathway, well known for its role in inflammation and immunity, is often constitutively activated in lung cancer. Engagement of CCR9/CCL25 or CXCL12/CXCR4 can trigger IκB degradation, leading to nuclear translocation of NF-κB subunits such as p65. This results in transcriptional activation of genes involved in proliferation (cyclin D1), anti-apoptosis (BCL-2, Survivin), and metastasis (MMP-9, VEGF). CXCR4 activation has also been shown to amplify NF-κB-mediated transcription via interaction with downstream intermediates such as TRAF6 and TAK1, promoting a pro-tumorigenic microenvironment (48, 49). Notably, NF-κB activation also upregulates CXCL12 and CCR9 expression, creating a positive feedback loop that sustains tumor-promoting inflammation and chemokine signaling.

## 3 Chemokines CCR9/CCL25 and CXCL12/CXCR4 in NSCLC

### 3.1 The prognostic impact of CCR9/CCL25 in NSCLC

The CCR9/CCL25 axis critically contributes to NSCLC progression by activating the PI3K/AKT pathway and serves as a robust prognostic indicator. Immunohistochemical analyses reveal consistent CCR9 overexpression in NSCLC tissues compared to adjacent non-tumorous and normal tissues, with particularly high levels observed in the squamous cell carcinoma line NCI-H157 relative to normal bronchial epithelial cells (40). Functional studies demonstrate that CCR9 silencing attenuates cancer cell proliferation and induces apoptosis, confirming its pro-tumorigenic role (24). Clinically, elevated CCR9 expression correlates with advanced tumor grade, lymph node metastasis (41), and poor survival, independent of histologic subtype (28, 34). Notably, CCR9 is more prevalent in adenocarcinomas than in squamous cell carcinomas (30), potentially explaining the former’s aggressive behavior and worse prognosis. This disparity may arise from histology-specific modulation of downstream effectors: adenocarcinomas exhibit higher serum CCL25 levels (20), while CCR9-driven upregulation of VEGF, MMP-2, and MMP-9 promotes angiogenesis and metastatic dissemination (41). Intriguingly, CCR9 also modulates anti-tumor immunity. Although peripheral CD4^+^ T cells from NSCLC patients show reduced CCR9 expression compared to healthy controls, post-surgical T cell populations exhibit CCR9 upregulation (42, 43). *In vitro*, CCL25 enhances CD4^+^; T cell migration, suggesting a dual role for this axis in both tumor progression and immune surveillance. Thus, assessing CCR9 and CCL25 expression may provide prognostic insights and improve postoperative immune function in NSCLC patients. Therefore, these chemokines serve as promising prognostic biomarkers and potential therapeutic targets, offering valuable insights for personalized treatment strategies in NSCLC and SCLC.

### 3.2 Biological properties of CXCL12/CXCR4 in relation to the tumor microenvironment

The tumor microenvironment (TME), comprising stromal and immune cells, plays a critical role in tumor formation, invasion, and (44–47). Chemokines act as central mediators within the TME, orchestrating the recruitment, spatial organization, and functional modulation of immune cell populations (48–51). Among these, CXCL12/CXCR4 signaling shapes a complex immunological landscape that supports tumor immune escape and progression (9, 52). T cells, particularly Tregs and exhausted CD8^+^; T cells, are recruited by the CXCL12 gradient in the TME, leading to an immunosuppressive milieu. Studies have shown that CXCL12-enriched zones can sequester effector T cells away from tumor nests, limiting cytotoxic responses (53). CXCR4 expression on CD8^+^; T cells are also associated with a dysfunctional phenotype characterized by reduced cytokine production and increased PD-1 expression (54).

The CXCL12/CXCR4 axis plays a multifaceted role in tumor progression through interactions with stromal and immune components. Vascular endothelial growth factor (VEGF) induces CXCL12-associated vascular gene expression in myofibroblasts, thereby recruiting monocyte-derived macrophages to target tissues and fostering a pro-tumorigenic microenvironment that supports tumor proliferation (55). In HER-2-positive breast cancer, HER-2 signaling upregulates CXCR4 expression while simultaneously inhibiting its degradation, a mechanism that contributes to enhanced invasive potential (56). Similarly, in lung cancer, elevated epidermal growth factor receptor (EGFR) expression has been tentatively linked to CXCR4 induction, correlating with poor clinical outcomes; however, further mechanistic validation is required to establish this relationship conclusively (56). The hypoxic tumor microenvironment further modulates CXCR4 activity, as HIF-1α drives CXCL12/CXCR4 upregulation under low oxygen conditions (57). Functionally, CXCR4 not only recruits immunosuppressive cells, such as neutrophils, tumor-associated macrophages, but also engages with transcription factors to activate the NF-κB pathway, collectively reshaping the TME to promote immune evasion and metastatic dissemination (58).

### 3.3 Association of CXCL12/CXCR4 with tumor growth

**Figure 1:**
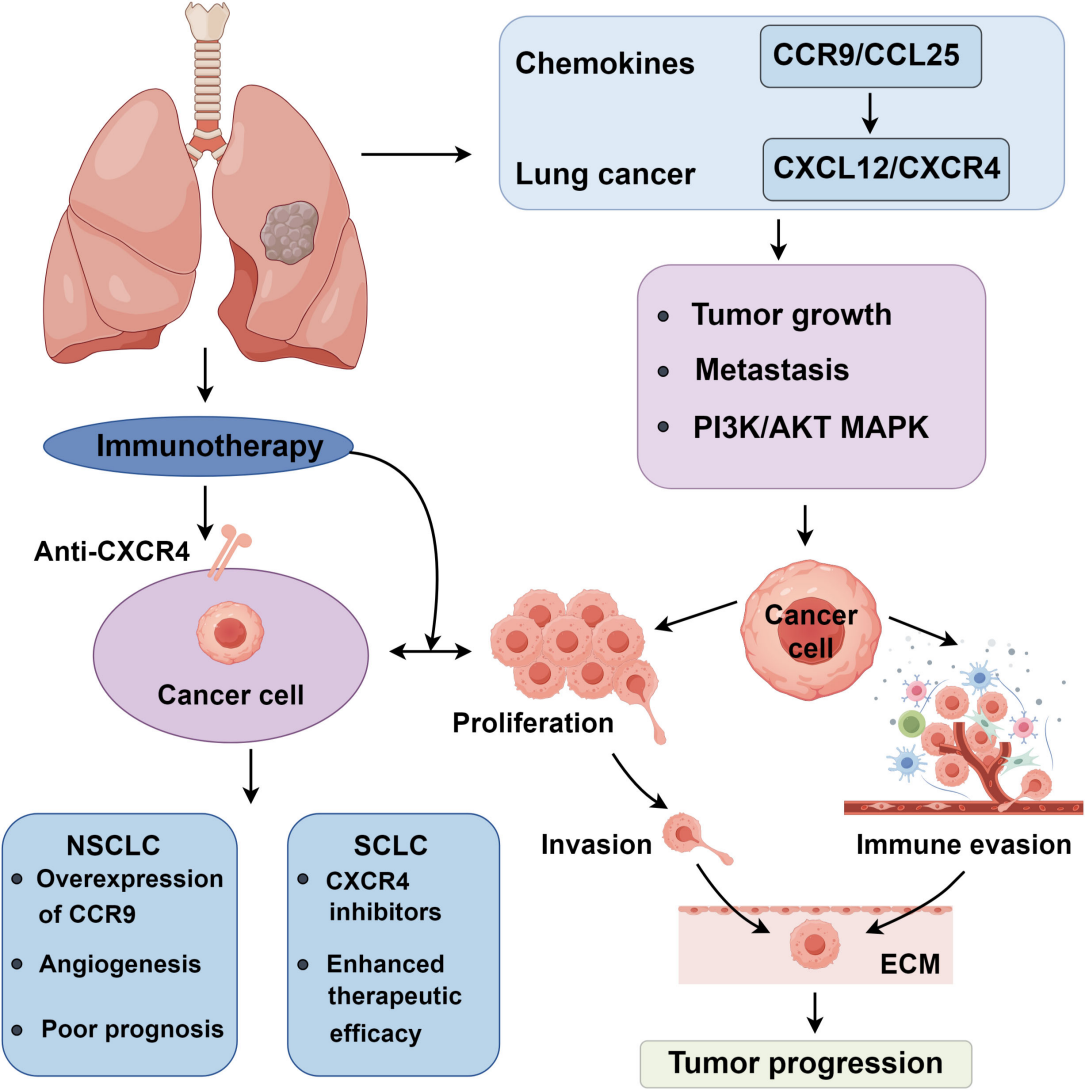
Roles of chemokines in lung cancer progression.

The unrestrained proliferation of tumor cells defines the essence of tumor growth, with proliferative signaling pathways often aberrantly activated within tumor cells. Studies highlight the critical role of CXCR4 in chronic lymphocytic leukemia by facilitating tumor migration. In triple-negative breast cancer, CXCR4 is crucial, operating via the LASP1-Ago2 axis. MicroRNA Let-7a can regulate the signaling of the CXCR4/LASP1 axis through competitive binding (59). Dąbrowska examined the expression of CXCL12 and CXCR4 in 100 breast cancer patients, revealing that analyzing the expression levels of CXCL12/CXCR4 can enhance the accuracy of breast cancer diagnosis (60). Research by Li suggests that CXCR4 is overexpressed in tumor tissues compared to normal tissues, with its expression correlating with B cell and CD8+ cell infiltration. Further studies associate high CXCR4 expression with poor prognosis in gastric cancer (58). Ottaviano et al. analyzed the relationship between CXCR4 expression and clinical parameters and prognosis in colon cancer, incorporating 78 patients. They found a 66.7% positivity rate for CXCR4 expression, which was prevalent in right-sided colon cancers and higher-grade tumors. Moreover, high CXCR4 expression could more effectively predict the efficacy of first-line treatments and was associated with worse outcomes in colon cancer patients (61) (**Figure 1**).

### 3.4 Expression and regulatory mechanisms of CXCR4 in SCLC

Small Cell Lung Cancer is highly malignant and rapidly progressive. CXCL12/CXCR4 plays a significant role in the oncogenesis and progression of tumors (62). Stumpf et al. analyzed 58 lung cancer patients, including cases of adenocarcinoma, squamous cell carcinoma, and SCLC, applying immunohistochemistry to assess CXCR4 protein expression. The results showed a 100% positivity rate for SCLC, 63.6% for adenocarcinoma, and 90.5% for squamous cell carcinoma. CXCR4 gene expression was closely linked to the expression of SST2 (63). Studies by Kijima et al. indicated high CXCR4 expression in SCLC cell lines, closely associated with tumor cell adhesion and migration, and correlated with p-AKT levels (64). Long non-coding RNAs (lncRNAs) also play a crucial role in regulating CXCR4 expression (65–67). LncRNA NORAD can downregulate CXCR4 via the RhoA/ROCK signaling pathway, inhibiting cell migration. LncRNA00922 accelerates lung cancer cell proliferation, invasion, and metastasis by regulating miRNA204/CXCR4. High CXCR4 expression in lung cancer correlates not only with cell invasion and metastasis but also with poor prognosis. Katsura et al. included 140 operable non-small cell lung cancer patients and found that high CXCR4 expression correlated with worse patient outcomes using immunohistochemical methods (68). Liang et al. included 2037 patients, showing that CXCR4 expression correlates with lymph node metastasis, distant metastasis, tumor staging, and survival, with higher expression associated with shorter survival (69). In summary, CXCR4 serves as a key driver of SCLC pathogenesis and advancement, positioning it as a potential candidate for targeted therapy in advanced disease.

### 3.5 Role of CXCR4 inhibitors in small cell lung cancer

The therapeutic landscape of lung cancer has evolved significantly with the identification of chemokine receptors as potential targets, particularly CXCR4 (70, 71). While CXCR4 inhibitors have shown promise in SCLC, emerging studies highlight their potential in NSCLC as well. In NSCLC, overexpression of CXCR4 is associated with poor prognosis and immune evasion (72). Recent studies indicate that CXCR4 expression increases with advancing NSCLC stages (73). Peptide R, a novel CXCR4 inhibitor, has demonstrated dual efficacy, it suppresses the dissemination of metastasis-initiating cells, enhances CD8^+^ T-cell activity, and reduces regulatory T-cell (Treg) infiltration in NSCLC models (74). These findings suggest its potential as a combinatorial therapeutic target to improve treatment outcomes. Additionally, co-delivery of miR-126-3p mimics and miR-221-3p inhibitors via lipid nanoparticles has been shown to inhibit tumor growth and metastasis by blocking AKT and CXCR4 signaling pathways (75). CXCR4 overexpression promotes tumor spheroid formation and epithelial-mesenchymal transition (EMT), whereas CXCR4-knockout murine models exhibit significantly smaller NSCLC tumor lesions compared to CXCR4-high counterparts (76).

In SCLC, progress in targeted therapies remains limited, but CXCR4 has emerged as a viable candidate. Salgia (77) conducted a Phase II study evaluating the efficacy of a CXCR4 inhibitor combined with etoposide and cisplatin in extensive-stage SCLC. This study included 90 patients and assessed CXCR4 expression in circulating tumor cells. Results indicated that combining a CXCR4 inhibitor with chemotherapy prolonged survival, with higher CXCR4 expression in circulating tumor cells predicting better treatment efficacy and longer disease-free and overall survival times. This suggests that CXCR4 inhibitors have potential as effective therapeutic targets in SCLC. A recent study demonstrated that targeting CXCR4 could enhance the efficacy of PD-1 immunotherapy by modulating the tumor microenvironment, primarily through the reduction of myeloid-derived suppressor cells. The COMBAT trial showed that a CXCR4 antagonist combined with PD-1 inhibitors and chemotherapy could improve disease control and prolong survival in pancreatic cancer, mainly through increased CD8+ cell infiltration and reduced myeloid-derived suppressor cells, suggesting that CXCR4 inhibitors combined with traditional therapies can improve prognosis for tumor patients (78, 79) (**Supplementary Table S1**).

## 4 Conclusion

Chemokines, particularly the CCR9/CCL25 and CXCL12/CXCR4 axes, play pivotal roles in lung cancer progression, immune cell recruitment, and tumor microenvironment remodeling. In NSCLC, CCR9/CCL25 drives proliferation, metastasis, and immune evasion through upregulation of VEGF, MMPs, and anti-apoptotic proteins, with its overexpression correlating with advanced disease and poor prognosis. Similarly, CXCL12/CXCR4 orchestrates immunosuppression by recruiting Tregs and MDSCs while promoting angiogenesis and EMT. In SCLC, CXCR4 is nearly ubiquitously expressed and linked to aggressive phenotypes, making it a compelling therapeutic target. Clinical studies highlight the potential of CXCR4 inhibitors to enhance chemotherapy and immunotherapy efficacy, as seen in the COMBAT trial, where CXCR4 blockade improved CD8^+^ T cell infiltration and reduced MDSCs. These findings underscore chemokines as dual-functional biomarkers for prognosis and promising targets for precision therapy.

Future research should prioritize translational applications, including the development of robust chemokine-based diagnostic panels and combinatorial regimens integrating CXCR4 inhibitors with immune checkpoint blockers or conventional therapies. Challenges such as off-target effects and optimal dosing require further investigation, particularly in SCLC, where treatment options remain limited. Additionally, exploring the crosstalk between chemokine networks and emerging resistance mechanisms could unveil novel therapeutic vulnerabilities. Clarifying the spatiotemporal dynamics of chemokine signaling across lung cancer subtypes may enable the development of subtype-specific therapeutic strategies, ultimately enhancing lung cancer patients’ survival and quality of life.
